# Tri-Trophic Impacts of Bt-Transgenic Maize on Parasitoid Size and Fluctuating Asymmetry in Native vs. Novel Host-Parasitoid Interactions in East Africa

**DOI:** 10.3390/insects9020038

**Published:** 2018-03-27

**Authors:** Dennis O. Ndolo, Josephine M. Songa, Gábor L. Lövei

**Affiliations:** 1 Biotechnology Centre, Kenya Agricultural Research Institute, P.O. Box 14733-00800 Nairobi, Kenya; dennis.ndolo@icgeb.org (D.O.N.); josephine.songa@gmail.com (J.M.S.); 2 Department of Agroecology, Aarhus University, Flakkebjerg Research Centre, DK-4200 Slagelse, Denmark

**Keywords:** GM plants, biosafety, natural enemies, morphological traits, environmental stress, stem borers, ecosystem services

## Abstract

Environmental stress can affect trait size and cause an increase in the fluctuating asymmetry (FA) of bilateral morphological traits in many animals. For insect parasitoids, feeding of hosts on transgenic maize, expressing a *Bacillus thuringiensis* toxin gene is a potential environmental stressor. We compared the size of antennae, forewings, and tibia, as well as their FA values, in two parasitoids developed on two East African host species feeding on non-transgenic vs. transgenic maize. The two lepidopteran stem-borer hosts were the native *Sesamia calamistis* Hampson (Lepidoptera: Noctuidae) and a recent invader, *Chilo partellus* Swinhoe (Lepidoptera: Crambidae). The two braconid parasitoids were the native, gregarious larval endoparasitoid *Cotesia sesamiae* and the recently introduced *Cotesia flavipes*. Both parasitoids attacked both hosts, creating evolutionarily old vs. novel interactions. Transient feeding of hosts on transgenic maize had various effects on FA, depending on trait as well as the host and parasitoid species. These effects were usually stronger in evolutionarily novel host–parasitoid associations than in the older, native ones. These parameters have capacity to more sensitively indicate the effects of potential stressors and merit further consideration.

## 1. Introduction

The use of genetic transformation technology in crops allowed several crop plants to express genes that encode insecticidal proteins (delta endotoxins) from the bacterium *Bacillus thuringiensis* Berliner. One of the first such plants is maize, *Zea mays* L., expressing the (activated) Bt toxin [1], because one of the main pests of maize in North America, the European corn borer, *Ostrinia nubilalis* Hubner (Lepidoptera: Pyralidae), is difficult to control by conventional pesticides, agronomic measures, or biological control agents [2]. Bt maize has been commercially grown in the USA since 1995 [3].

Maize is an important staple crop in sub-Saharan Africa (SSA), and is much affected by insect pests there [4]. In Africa, insect-resistant Bt maize is commercially grown only in South Africa [5], though several projects involving transgenic maize are now underway. In Kenya, the Insect Resistant Maize for Africa (IRMA) Project has been evaluating some varieties of transgenic Bt maize for eventual introduction [6].

The procedure before a field release of transgenic crop requires an environmental risk assessment [7]. Kenya is among the first countries in Africa with an existing biosafety law where this requirement is included [8]. The ultimate reason for biosafety tests is that agricultural innovations should not put further pressure on ecosystem services [9] that are vitally important for humankind [10] and are under increasing pressure world-wide [11]. This is of special relevance in Africa, where farmers depend more on ecosystem services than those in developed countries [12].

Natural pest control is one such ecosystem service [13] and there is experimental evidence that genetically modified (GM) plants can have non-neutral impacts on natural enemies, including predators and parasitoids [14]. A summary of the available data on natural enemies of pests [15] indicates a lack of data from Africa.

Insect-resistant transgenic plants may kill immature parasitoids indirectly by killing their host [16], or by rendering the host nutritionally inferior or unsuitable [17]. Parasitoids can also be sensitive to changes in nectar composition [18] or volatile profile [19,20] that occur in transgenic plants. They are also sensitive to host quality, which can be influenced by host plants, giving rise to modified tri-trophic interactions [21]. When the host feeds on Bt-containing food, the sex ratio of its parasitoid can also change [22].

Tests of the impact of transgenic plants on natural enemies usually measure life history variables such as growth, mortality, or development time. In a few experiments, behavioral [23] or physiological [24] parameters were also measured. Conditions during development, and the fitness of the emerging organism, could also be characterized by checking different morphological features. Many insect body parts have two copies, and under ideal conditions, these should display perfect symmetry. However, such features are rarely in perfect symmetry [25]. Deviations can be unidirectional (directional asymmetry, [26]) or can deviate in either direction (fluctuating asymmetry). Fluctuating asymmetry (FA) refers to random deviations from symmetry of otherwise bilaterally symmetric traits, and is supposed to occur when an individual is unable to undergo identical development on the two sides of a bilaterally symmetrical morphological trait [27]. Environmental stress can cause an increase in the FA of morphological traits [27] and the degree of increase has been used as a measure of the seriousness of such stress [27,28,29]. Extreme temperatures [30,31], exposure to pesticides [32,33], and suboptimal food quality [34] or quantity [26] could increase FA in morphological traits during development.

Generally, there is a negative correlation between the degree of FA and population fitness. For instance, the lifespan of *Malacosoma disstria* Hubner (Lepidoptera: Lasiocampidae) was shortened as the degree of FA of the first segment of foreleg tarsi increased [35]. Furthermore, there is a positive association between adult body size and standard measures of fitness under both laboratory and field conditions [36,37]. In Hymenoptera, antennal, forewing, and tibia length are good indicators of quality/fitness [37].

Parasitoid size and fitness may be positively or negatively correlated to host condition, which, in turn, can be strongly dependent on the quantity and quality of food [36]. Bt-intoxicated *Eoreuma loftini* Dyar (Lepidoptera: Crambidae) had significant negative effects on certain fitness parameters of the parasitoid *Parallorhogas pyralophagus* Marsh (Hymenoptera: Braconidae) [38], as did Bt-intoxicated *Spodoptera frugiperda* (Lepidoptera: Noctuidae) on its braconid parasitoid *Cotesia marginiventris* Cresson (Hymenoptera: Braconidae) [39].

In our laboratory experiments, we hypothesized that feeding on transgenic Bt maize would constitute a feeding stress for lepidopteran larvae, and their quality as hosts for the parasitoid would decrease. We expected that this would influence morphological parameters such as body size, as well as cause an increase in FA.

We found that transient feeding on transgenic Bt maize had a negative effect on some body size parameters and, to a lesser degree, on FA in two hymenopteran parasitoids kept on two species of African stem borers, and these effects were stronger in the evolutionarily novel host–parasitoid associations than in the evolutionarily older, native ones.

## 2. Materials and Methods

### 2.1. Plant Material

Plant material was obtained from Event 216 (Bt maize) (described in [40]). The isogenic line CML 216 [6] was used as a non-transgenic control. These plants were grown in 30 cm diameter pots in a biosafety greenhouse at the Kenya Agricultural Research Institute (KARI), Nairobi, Kenya, at temperatures of approximately 25 °C and natural light conditions of approximately 12L:12D photoperiod. Before use, pieces of plant material were washed in a 2% solution of commercially available bleach (0.05% sodium hypochlorite) to kill any microbial contaminants originating from the greenhouse, rinsed in distilled water, and dried.

#### 2.1.1. Stem Borers

Two species of African stem borers were used as hosts: *Chilo partellus* Swinhoe (Lepidoptera: Crambidae) and *Sesamia calamistis* Hampson (Lepidoptera: Noctuidae). Both are among the most damaging stem borer species in Kenya [41]. *C. partellus* is an Asian species introduced to East Africa in the early 1930s [42], while *S. calamistis* is a native stem borer species that occurs in all areas of East Africa up to 2400 m above sea level [3].

The *C. partellus* and *S. calamistis* larvae used in this study were obtained as eggs from the insectaries at the International Centre of Insect Physiology and Ecology (ICIPE), Nairobi, Kenya and KARI, Katumani Centre, Machakos, Kenya. Both originated from colonies maintained on artificial diet of Ochieng et al. [43]. The eggs were incubated in 700-mL jam jars in the laboratory at the National Agricultural Research Laboratories of the Kenya Agricultural Research Institute (KARI), Kabete, Nairobi, at 25 ± 1 °C and 12 h/12 h; light/dark photoperiod. Stem borer larvae were kept on pieces of the non-transgenic plant material in a 700-mL jam jar, lined with moist filter paper and perforated lids (to allow for air circulation) until fourth instar, when they were used for the experiment. First to third instars were fed on maize leaf material, while later instars were fed on maize stems. Leaf pieces were changed every two days, and stem pieces every 3–4 days, when the filter papers were also re-moistened with distilled water. The moist filter paper was meant to keep the plant material fresh for longer.

### 2.2. Parasitoids

Two larval parasitoids were selected as natural enemies. The gregarious larval endoparasitoid *Cotesia sesamiae* Cameron (Hymenoptera: Braconidae) is an indigenous species that attacks mid- to late-instar stem borer larvae [44], and is the most widespread larval parasitoid in eastern parts of Kenya [45]. *Cotesia flavipes* Cameron (Hymenoptera: Braconidae) is an introduced species, attacking both *C. partellus* and *S. calamistis* [46,47]. This is also a gregarious species, and is closely related to *C. sesamiae* [46]. Parasitized host larvae continue to feed during the development of the parasitoid larvae, and thus the quality of the host diet can affect parasitoid development [48].

The parasitoids used in this study originated from a laboratory-reared population maintained at the International Centre for Insect Physiology and Ecology, Nairobi, Kenya. These colonies were periodically “refreshed” by introducing field collected adults to maintain genetic diversity in the rearing. The parasitoids were reared on a combination of *S. calamisits* and *Chilo partellus* to mimic what would prevail in nature, where they would be exposed to different parasitoids as the stem borers occur in complexes with overlapping spatial and temporal distributions [41]. Parasitoids were kept at approximately 27 °C, 65–70% RH and a 12 h:12 h L:D photoperiod [41]. Newly emerged adult parasitoids were kept in 20 cm^3^ Perspex cages and provided with a 20% honey solution; they were left to mate for 24 h prior to host exposure.

### 2.3. Experimental Setup

Fourth-instar larvae of each of the two stem borer species were subjected to transient feeding on Bt maize for 24 h.To enhance feeding on Bt maize (toxin ingestion), the larvae were starved for 24 h prior to exposure to the treatments. The larvae were transferred individually onto pieces of plant material in moist filter paper. After 24 h, the larvae were held individually using forceps and exposed to 24 h-old mated female parasitoids in Perspex cages. The parasitoids readily attacked the host and usually oviposited in a few seconds, after which the host larvae were individually placed on diet containing non-transgenic plant material [41]. This treatment intended to simulate field situations where stem borer larvae move off Bt maize after trial feeding. Such ‘transient shocks’ are suitable because they bring out the possible impact of a stress factor on FA [49]. Bt-induced mortality of such transient feeding on non-parasitized hosts was approximately 45% and 41% for *C. partellus* and *S. calamistis,* respectively [50]. Each parasitoid species was exposed to i) each of the stem borer species exposed to Bt maize and ii) each of the stem borer species reared on non-transgenic maize (Table 1).

Parasitized larvae were reared in Petri dishes on pieces of fresh, non-transgenic control maize stems (which were changed every 3–4 days) and inspected daily until parasitoid cocoon production. Emerging cocoons were collected separately from each larva, and incubated in the laboratory in transparent glass vials stopped with cotton wool.

Twenty female wasps were randomly selected from each treatment and measured using Leica Application Suite Educational Zoom (LAS EZ, Microsoft Corporation) software. Three traits were measured (±0.01 µm) three times on both sides: hind tibia length (measured from the femur/tibia joint to the tibia/tarsus joint), wing length, and antennal length. These traits are good indicators of the field quality of hymenopteran wasps [37].

The FA value was calculated using the formula [51]:

FA=mean {(|L−R|)/ (L+R)/2},(1)

where L and R represent the left and right length of a trait, respectively.

### 2.4. Data Evaluation

Analysis of variance was used to compare FA of *C. flavipes* and *C. sesamiae* among the larvae subjected to transient feeding on Bt maize and those reared exclusively on non-Bt maize. When ANOVA indicated significant (*p* < 0.05) differences, Student–Newman–Keuls (SNK) tests were used to identify treatments that were different.

Additionally, in an approach akin to that adopted by Lövei et al. [14], we assessed the overall response direction for each of the parameters across the host and parasitoid species tested.

## 3. Results

### 3.1. Antennal Length, Wing Length, and Hind Tibia Length

Wing length in parasitoids was sensitive to host plant effects: in all combinations but one, wing lengths were significantly lower on parasitoids developing on hosts subjected to transient feeding on Bt maize than on the control (*F* = 49.3, d.f. = 1.38, *p* = 0.0005 for *C. flavipes* on *C. partellus*; *F* = 5.7, d.f. = 1.38, *p* = 0.022 for *C. sesamiae* on *C. partellus*; *F* = 13.6, d.f. = 1.38, *p* = 0.001 for *C. flavipes* on *S. calamistis*). The reduction in wing length in *C. sesamiae* developed in *S. calamistis* was non-significant (Table 1).

Only two of the host–parasitoid–host plant combinations caused significant changes in antennal length: *C. flavipes* developing on Bt-fed *C. partellus* hosts had significantly reduced antennal length (Table 1, *F* = 53.9, d.f. = 1.38, *p* = 0.0005). Conversely, antennal length was significantly increased in *C. sesamiae* developing on Bt-fed *S. calamistis* hosts (*F* = 7.7, d.f. = 1.38, *p* = 0.009). For the two “evolutionarily novel host-parasitoid combinations” (*C. flavipes* on *S. calamistis* and *C. sesamiae* on *C. partellus*), there was a non-significant decrease.

Hind tibia lengths were significantly reduced in the two evolutionarily novel combinations: *C. flavipes* developing on Bt-fed *S. calamistis* (*F* = 14.1, d.f. = 1, 38, *p* = 0.001) and in *C. sesamiae* developing on Bt-fed *C. partellus* (*F* = 40.0, d.f. = 1.38, *p* = 0.0001, Table 1). Similarly to the antennal length, non-significant reductions in tibia length were measured in the other two species combinations as well (Table 1).

### 3.2. Fluctuating Asymmetry in Antennal Length, Wing Length, and Tibia Length

FA values of wing length was significantly different only in the *C. flavipes/S. calamistis* combination, where wasps emerging from Bt-feeding hosts had significantly higher FA in wing length (*F* = 8.8, d.f. = 1.38, *p* = 0.005, Table 2). There was a non-significant increase for *C. sesamiae*/*S. calamistis* and non-significant reductions for *C. flavipes/C. partellus* and *C. sesamiae/C. partellus.*

FA values for hind tibia length of *C. flavipes* developing on *S. calamistis* were not significantly affected by transient feeding of the hosts on Bt maize. However, *C. sesamiae* developing on Bt-fed *C. partellus* had significantly higher (*F* = 40.0, d.f. = 1, 38, *p* = 0.0005) FA values compared to those developing on non-Bt maize-fed hosts (Table 2). For *C. flavipes* / *S. calamistis* there was a non-significant decrease, while for *C. flavipes*/*C. partellus* and *C.sesamiae*/*S. calamistis* there was a non-significant increase on Bt maize-fed hosts.

*C. flavipes* did not demonstrate significant differences in antennal FA in response to host feeding (Table 2). *C. sesamiae* was more sensitive: when developing on Bt-fed novel host, *C. partellus*, emerging adult wasps had significantly higher FA in antennal length (*F* = 54.0, d.f. = 1, 38, *p* = 0.0005) compared to those developing on the non-transgenic control. Conversely, when the same parasitoid was developing on its Bt-fed original host, *S. calamistis*, the adult parasitoids had significantly lower FA values (*F* = 82.5, d.f. = 1.38, *p* = 0.0005) compared to the non-transgenic control (Table 2).

Most response parameters were negatively affected by feeding on Bt transgenic maize, except for antennal measurements, which exhibited a significantly positive response in terms of both length and FA in two cases (Table 3).

## 4. Discussion

The experimental setup with the hosts was intended to simulate field situations where stem borer larvae move between different maize plants, or leave Bt maize plants after trial feeding. Previous studies have mainly used sublethal Bt toxin concentrations with continuous, rather than partial exposure [52]. In reality, susceptible insects exposed continuously to Bt plants invariably suffer complete mortality; only those insects subjected to partial feeding on the Bt plants have high chances of survival. Target arthropods tend to avoid the toxins present in GM crops and display escape behavior [53]. Continuous exposure to sublethal Bt toxin concentrations therefore does not capture the actual situation in the field. Due to resistance management practices [54], patches of non-transgenic maize plants coexist alongside genetically modified (GM) plants. Several maize herbivores, such as stem borer larvae, especially later instars, can move between host plants [55,56,57,58].

Overall, our results confirmed the importance of host quality for parasitoids. They also indicate that host feeding on transgenic Bt constitutes an additional stress for parasitoids, and more so in evolutionarily novel parasitoid–host combinations than in evolutionarily older, native ones. This stress manifested itself in several morphological characteristics that are related to fitness.

Wing length is extremely important in hymenopterans as it affects flight ability [59]. Hind tibia and antennal length are reliable estimators of body size in insects [37,60,61]. There is often a positive association between adult body size and standard laboratory and field fitness measures [36,37]. Wing asymmetry could influence flight ability, which could affect the ability of parasitoids to reach hosts [37]. Since hind tibia length and antennal length are correlated to insect size and hence fitness, asymmetry in these traits could also possibly affect insect fitness.

Wing length in all host–parasitoid combinations was reduced following transient host feeding on Bt maize. The reduction was not significant for *C. sesamiae* developing on *S. calamistis* hosts. *C. sesamiae* and *S. calamistis* are both native species in East Africa, and it is feasible that *S. calamistis* has, as a result of its long association with the parasitoid, developed mechanisms to overcome attack by the parasitoid. Encapsulation of *C. sesamiae* eggs in *S. calamistis* occurs [62,63] but success of such defense depends on herbivore vigor [64], which can be reduced by host-plant-induced stresses [65,66,67]. The usual successful prasitisation (cocoon formation) of *C. sesamiae* on *S. calamistis* is 55% [68]. The observed positive effects of Bt intoxicated *S. calamistis* larvae on *C. sesamiae* [69] were attributed to a weakened host immune system, resulting in a lower encapsulation rate of the parasitoids’ eggs by the host larva. The parasitoid *Cotesia kazak* Telenga (Hymenoptera: Braconidae) has more success on its host *Helicoverpa armigera* Hubner (Lepidoptera: Noctuidae), fed on less toxic Bt-amended diets [70], as does *Tranosema rostrale* Brishke (Hymenoptera: Ichneumonidae) developing on the Bt-fed spruce budworm *Choristoneura fumiferana* Clemens (Lepidoptera: Tortricidae) [71].

The shorter parasitoid wings in the other host–parasitoid combinations were possibly due to the novelty of these associations. The ichneumonid *Venturia canescens* Gravenhorst (Hymenoptera: Ichneumonidae), when developing in Bt fed *Plodia interpunctella* Hubner (Pyralidae: Phyctinae), also develops shorter wings [72]. *C. flavipes* developing on *S. calamistis* was most affected: both wing length and its FA were significantly and negatively affected when the parasitoid was on Bt-fed hosts. *S. calamistis* is not a very good host for *C. flavipes* [73,74] and, apparently, Bt-feeding makes it even less suitable.

Hind tibia lengths in parasitoids were reduced following host exposure to Bt maize, with the reduction being significant for the evolutionarily novel combinations: *C. flavipes* developing on *S. calamistis* and *C. sesamiae* developing on *C. partellus*. The lack of significant effects on tibial length, as well as FA in *C. sesamiae* developing on *S. calamistis*, could have resulted from the weakening of the host’s defenses by the Bt toxin. As *S. calamistis* is not a very suitable host for *C. flavipes*, the additional stressor, whether in the form of the Bt toxin or biochemical changes in the host, could contribute to the negative impact. The changes in FA for tibia were not significant in any host–parasitoid combinations, indicating that hind tibia symmetry is either unaffected or not sensitive to stress factors emerging from hosts feeding on Bt maize.

Antennal length was significantly increased in *C. sesamiae* developing on *S. calamistis* larvae (possibly due to the reasons mentioned earlier). In the *C. flavipes/C. partellus* combination, it was significantly reduced, possibly due to the evolutionarily novel host–parasitoid association.

The lack of consistency in FA responses across traits may reflect the variation between trait types in their susceptibility to environmental stress [75]. In most cases, the effect of Bt-fed hosts on these parasitoids was negative. This reinforces the higher sensitivity of parasitoids to host quality than that of predators to prey quality [15]. Our experiments used realistic scenarios (variable temperatures and host plant, not diet used), and overcame many of the traditional problems of previous “non-target” tests [14]. However, the studied parameters, while related to fitness, do not predict the amount of fitness change. They, however, have the potential to more sensitively indicate the effects of potential stressors and merit further consideration and more detailed study. In assessing the risks posed to non-target organisms by transgenic plants, it would be desirable to quantify the relationship between the FA values and fitness parameters of the studied organisms.

By studying evolutionarily “old” vs. “novel” host–parasitoid associations, we found that parasitoids in novel associations were more sensitive to host quality, lowered by the Bt treatment, and can be negatively affected. These results underline the importance of considering multiple factors when assessing the impact of genetically modified plants on ecosystem service providers [76]. The parameters used here reflect parasitoid quality well, have shorter time lags than other, traditional population parameters, and have the potential to move pre-release risk assessment tests to become more realistic ecologically [14].

Factors that negatively impact on fitness of such biocontrol agents could result in a lower level of ecosystem services. The effect on these ecological processes, which operate on vast scales and from which we derive substantial benefits, should be evaluated when harmonization of the GM technology and biological control is sought [77].

## Figures and Tables

**Table 1:** Antennal length, wing length, and hind tibia length (Mean ± SE) of the parasitoids *Cotesia flavipes* and *Cotesia sesamiae* developed on the stem borers *Chilo partellus* and *Sesamia calamistis* on Bt maize vs. non-Bt maize for 24 h (*n* = 20 each).

Parasitoid, Host, and Host Feeding Regime	Antennal Length (μm)	Wing Length (μm)	Hind Tibia Length (μm)
*C. flavipes* on *C. partellus*			
fed on non-Bt	1110.8 ± 15.0 ^a^ #	782.3 ± 10.4 ^a^	486.8 ± 8.9 ^a^
fed on Bt	954.7 ± 15.0 ^b^	678.8 ± 10.4 ^b^	466.4 ± 8.9 ^a^
*C. flavipes* on *S. calamistis*			
fed on non-Bt	1084.3 ± 28.4 ^a^	792.4 ± 13.9 ^a^	547.6 ± 12.2 ^a^
fed on Bt	1035.9 ± 28.4 ^a^	720.0 ± 13.9 ^b^	482.9 ± 12.2 ^b^
*C. sesamiae* on *C. partellus*			
fed on non-Bt	995.2 ± 21.0 ^a^	795.5 ± 15.9 ^a^	544.4 ± 9.0 ^a^
fed on Bt	1015.5 ± 21.0 ^a^	741.7 ± 15.9 ^b^	463.8 ± 9.0 ^b^
*C. sesamiae* on *S. calamistis*			
fed on non-Bt	989.0 ± 20.4 ^a^	804.5 ± 14.4 ^a^	469.8 ± 10.9 ^a^
fed on Bt	1068.9 ± 20.4 ^b^	750.7 ± 14.4 ^a^	467.6 ± 8.2 ^a^

# Within a column, values with different superscripts are significantly different at *p* < 0.05 using the Student-Newman-Keuls test.

**Table 2:** Fluctuating asymmetry (Mean ± SE) in antennal length, wing length, and hind tibia length of the parasitoids *Cotesia flavipes* and *Cotesia sesamiae* developed on stem borer hosts *Chilo partellus* and *Sesamia calamistis* subjected to transient feeding on Bt maize (*n* = 20 in each combination).

Parasitoid, Host, and Host Feeding Regime	Fluctuating Asymmetry Values		
	Antennal Length	Wing Length	Hind Tibia Length
C. flavipes on C. partellus			
fed on non-Bt maize	0.039 ± 0.011 ^a^ #	0.051 ± 0.013 ^a^	0.105 ± 0.026 ^a^
fed on Bt maize	0.078 ± 0.019 ^a^	0.024 ± 0.013 ^a^	0.173 ± 0.026 ^a^
C. flavipes on S. calamistis			
fed on non-Bt maize	0.078 ± 0.019 ^a^	0.018 ± 0.003 ^a^	0.097 ± 0.016 ^a^
fed on Bt maize	0.090 ± 0.019 ^a^	0.032 ± 0.003 ^b^	0.054 ± 0.016 ^a^
C. sesamiae on C. partellus			
fed on non-Bt maize	0.038 ± 0.008 ^b^	0.072 ± 0.014 ^a^	0.055 ± 0.013 ^b^
fed on Bt maize	0.122 ± 0.008 ^a^	0.048 ± 0.014 ^a^	0.092 ± 0.013 ^a^
C. sesamiae on S. calamistis			
fed on non-Bt maize	0.124 ± 0.007 ^a^	0.070 ± 0.019 ^a^	0.055 ± 0.013 ^a^
fed on Bt maize	0.032 ± 0.007 ^b^	0.072 ± 0.019 ^a^	0.091 ± 0.013 ^a^

# Within a column, numbers with different superscripts indicate a significant difference at *p* < 0.05 using the Student-Newman-Keuls test.

**Table 3:** Summary of the response parameters (percentages) of the effects of transgenic maize containing Cry1Ab on parasitoids of two African stem borer species. Increase in fluctuating asymmetry was considered a negative consequence (number of cases in parentheses).

Trait	Measurement	Negative, Significant	Negative, Not Significant	Positive, Not Significant	Positive, Significant
Wing	Length (μm)	75 (3)	25 (1)	0	0
	Fluctuating Asymmetry	25 (1)	50(2)	0	25 (1)
Tibia	Length (μm)	50 (2)	50 (2)	0	0
	Fluctuating Asymmetry	25 (1)	50 (2)	0	25 (1)
Antennae	Length (μm)	25 (1)	25 (1)	25 (1)	25 (1)
	Fluctuating Asymmetry	25 (1)	50 (2)	25 (1)	0
